# 0896. Temporal changes in tidal recruitment compared to histologic outcome in experimental acute respiratory distress syndrome

**DOI:** 10.1186/2197-425X-2-S1-O22

**Published:** 2014-09-26

**Authors:** J Haase, S Hammermüller, A Beilicke, P Spieth, D Buchloh, S Haska, K Noreikat, T Muders, A Reske, H Wrigge

**Affiliations:** University of Leipzig, Anesthesiology and Intensive Care Medicine, Leipzig, Germany; University of Dresden, Department of Anesthesiology and Intensive Care Medicine, Dresden, Germany; University of Bonn, Department of Anesthesiology and Intensive Care Medicine, Bonn, Germany

## Introduction

Acute Respiratory Distress Syndrome (ARDS) and Ventilator-Associated Lung Injury (VALI) are characterized histologically by Diffuse Alveolar Damage (DAD)^1^. Tidal recruitment (TR) of nonaerated airspaces during mechanical ventilation (MV) causes VALI. Different MV strategies aim to reduce TR and VALI by individualized PEEP^2-4^.

## Objectives

For 3 MV strategies we quantified TR by computed tomography (CT) and electrical impedance tomography (EIT) as well as their association with DAD.

## Methods

After approval by the animal welfare committee, pigs underwent anesthesia, tracheostomy, MV and ARDS induction by tracheal hydrochlorid acid installation. MV was randomized according to the ARDS network lower PEEP (ARDSnet, n=8), Open Lung Concept (OLC, n=8) or EIT (n=10) protocols. In ARDSnet PEEP was set according to the table. In groups OLC and RVD, recruitment maneuvers preceded decremental PEEP titration. In OLC the PEEP at which the highest individual PaO_2_/FiO_2_ dropped by 10% was chosen. The minimal Regional Ventilation Delay Index (RVD) determined during a low-flow maneuver defined the best PEEP step in the EIT group^4^. TR was measured every 4 hours as the difference in nonaerated tissue between expiratory and inspiratory CT. The RVD was used to set PEEP only in the EIT group, but it was measured every 4 hours to assess heterogeneous lung aeration in all groups. TR- and RVD-data were multiplied by the time difference to the last measurement and cumulatively expressed as TRhours and RVDhours over 24 hours. Lung tissue samples from the left lower lobe were stained with hematoxylin/eosin. The DAD score for intraalveolar edema, inflammatory infiltration and hemorrhage was calculated as mean of 3 blinded investigators. ANOVA (Sidak's post-hoc) and linear regression were used. Results are presented as mean±SD.

## Results

**Figure 1:**
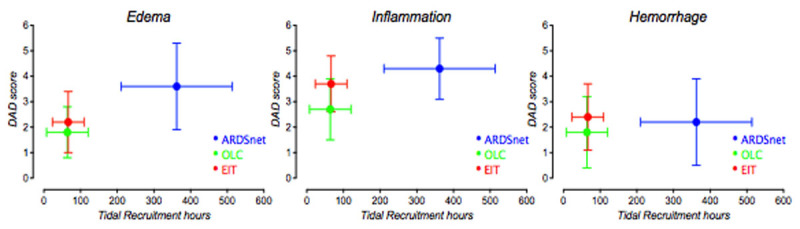
Figure 1

Figure 1 summarizes the results. TRhours were significantly higher in ARDSnet (362±152) than in OLC (70±57) and RVD (65±45). RVDhours showed the same behavior (ARDSnet 211±53, OLC 120±30, RVD 104±40) (both P< 0.0001). Correlation between TRhours and RVDhours was strong (R^2^=0.7, P< 0.0001). The edema score of ARDSnet (3.6±1.7) significantly exceeded OLC (1.6±0.9, P=0.04), but not RVD (2.2±1.3, P=0.13). Inflammation scores were also higher in ARDSnet (4.3±1.1) than OLC (2.5±1.1, P=0.03), but not higher than RVD (3.6 ±1.1, P=0.7). OLC and RVD differed neither for edema nor for inflammation. Hemorrhage did not differ between groups (P=0.68). DAD scores did not increase significantly across quintiles of TRhours or RVDhours (all P>0.16).

## Conclusions

MV according to ARDSnet is associated with more TR, greater ventilation inhomogeneity and increased histological DAD scores compared to OLC or EIT-RVD approaches. The association of TR surrogates assessed by CT or EIT with histological DAD features warrants further studies.
